# Eco-anxiety, how can the awareness on fighting global warming is becoming a mental health problem

**DOI:** 10.1192/j.eurpsy.2024.980

**Published:** 2024-08-27

**Authors:** M. Barbosa, A. R. Fonseca

**Affiliations:** SPSM, Centro Hospitalar de Leiria, Leiria, Portugal

## Abstract

**Introduction:**

Although the issue of climate change usually brings thoughts of environmental impact and physical health concerns to our consciousness, climate change also affects people’s mental health. Nowadays there is an emerging condition about climate change anxiety (CCA), defined as negative responses associated with global warming, with apprehension and stress related to the anticipation of threats to the ecosystem and our species. It may include cognitive, emotional, and behavioral responses, for example, persistent worries, psychological distress, or sleep difficulties related to long-term consequences of climate change, and can result in functional impairment.

**Objectives:**

A literature review to analyse the evidence, to be aware of individuals overconcerned about global warming, bring awereness and promote an appropriate seek of professional help when needed

**Methods:**

Using the Medline database through the Pubmed search engine was used, with the keywords: “climate change anxiety”, “eco-anxiety”.

**Results:**

Despite the lack of studies, CCA affects a substantial proportion, especially the younger population, aged from 16 to 25 years old worldwide. As a result of ecoanxiety, people are becoming anxious about their future and the future of the planet as we currently know it, the terrifying and at some extent uncertainty of it. It is documented the importance of green and blue spaces (water places) specially in urban areas for mental wellbeing – as our environment is quickly changing with the global warming, the reduction/disappearing of these areas are ongoing; besides this direct consequence, the disruption of these places results in feelings of loss due to changes to personally significant places a phenomenon known as ‘ecological grief’. Additionally, the occurrence of natural disasters like heatwaves, hurricanes, flooding, wildfire, and drought, raising concern and the socially-mediated impacts of forced migration and conflict caused by it. Self-reported presentations may include panic attacks, insomnia, obsessive thinking, and/or appetite changes caused by environmental concerns. If prolonged symptoms, depressive, anxious disease, post-traumatic stress disorder, among others can develop.

**Conclusions:**

To reduce eco-anxiety individuals can take steps to reduce their carbon footprint, engage in activism and advocacy, bringing more awareness to the subjects and thus taking measures to mitigate the effects of climate change and protect the environment. It’s equal important to consider and address the mental health impacts of climate change, this additionally includes providing adequate emotional and psychological support to those affected.

**Disclosure of Interest:**

None Declared

